# Machine learning for maternal health: Predicting delivery location in a community health worker program in Zanzibar

**DOI:** 10.3389/fdgth.2022.855236

**Published:** 2022-08-17

**Authors:** Alma Fredriksson, Isabel R. Fulcher, Allyson L. Russell, Tracey Li, Yi-Ting Tsai, Samira S. Seif, Rose N. Mpembeni, Bethany Hedt-Gauthier

**Affiliations:** ^1^ Department of Biostatistics, Harvard T.H. Chan School of Public Health, Boston, MA, United States; ^2^ Department of Global Health and Social Medicine, Harvard Medical School, Boston, MA, United States; ^3^ Harvard Data Science Initiative, Cambridge, MA, United States; ^4^ D-tree International, Dar es Salaam, Tanzania; ^5^ Department of Epidemiology and Biostatistics, Muhimbili University of Health and Allied Sciences, Dar es Salaam, Tanzania

**Keywords:** maternal health, machine learning, digital health, global health, facility delivery, community health worker intervention, artificial intelligence

## Abstract

**Background:**

Maternal and neonatal health outcomes in low- and middle-income countries (LMICs) have improved over the last two decades. However, many pregnant women still deliver at home, which increases the health risks for both the mother and the child. Community health worker programs have been broadly employed in LMICs to connect women to antenatal care and delivery locations. More recently, employment of digital tools in maternal health programs have resulted in better care delivery and served as a routine mode of data collection. Despite the availability of rich, patient-level data within these digital tools, there has been limited utilization of this type of data to inform program delivery in LMICs.

**Methods:**

We use program data from 38,787 women enrolled in Safer Deliveries*,* a community health worker program in Zanzibar, to build a generalizable prediction model that accurately predicts whether a newly enrolled pregnant woman will deliver in a health facility. We use information collected during the enrollment visit, including demographic data, health characteristics and current pregnancy information. We apply four machine learning methods: logistic regression, LASSO regularized logistic regression, random forest and an artificial neural network; and three sampling techniques to address the imbalanced data: undersampling of facility deliveries, oversampling of home deliveries and addition of synthetic home deliveries using SMOTE.

**Results:**

Our models correctly predicted the delivery location for 68%–77% of the women in the test set, with slightly higher accuracy when predicting facility delivery versus home delivery. A random forest model with a balanced training set created using undersampling of existing facility deliveries accurately identified 74.4% of women delivering at home.

**Conclusions:**

This model can provide a “real-time” prediction of the delivery location for new maternal health program enrollees and may enable early provision of extra support for individuals at risk of not delivering in a health facility, which has potential to improve health outcomes for both mothers and their newborns. The framework presented here is applicable in other contexts and the selection of input features can easily be adapted to match data availability and other outcomes, both within and beyond maternal health.

## Introduction

Maternal and neonatal health outcomes in low- and middle-income countries (LMICs) have improved over the last two decades, in part due to increasing access to facilities for delivery and the quality of care therein (1–3). Facility-based delivery facilitates timely access to emergency obstetric care, and higher rates of facility delivery in sub-Saharan Africa (SSA) are significantly associated with lower maternal mortality (4–6). Between 2010 and 2018, it is estimated that nearly 67% of women in SSA delivered in health facilities (7); however, many pregnant women still deliver at home in LMICs (8), increasing the risk of poor outcomes for both the mother and the child should a complication occur during childbirth. Strategies to identify and target interventions for these women could help close the remaining gap.

Previous research exploring the likelihood of facility-based delivery in SSA has found that attendance of at least one antenatal care visit, proximity to a health facility, difficulty delivering in the past, lower parity, higher maternal education and partner involvement reduces the likelihood of home delivery (9–14). Further studies suggest that local tradition and beliefs, cost of delivery, household income, perceived quality of care and fear of discrimination are also important factors influencing the choice of delivery location (15–20). This literature provides crucial insights into strong predictors of health facility delivery but does not maximize prediction accuracy for the purposes of real-time programmatic use.

The uptick of digital tools in maternal health programs, such as mobile devices for community health workers (CHWs) (21–26), presents a unique opportunity for real-time implementation of predictive models (23). Digital tools capture data of individuals enrolled in these programs that can be used to build prediction models (24); specifically, for maternal health, machine learning techniques can capture complex, non-linear relationships between demographic or clinical characteristics of the woman and birth outcomes (27–29). These machine learning models could then be applied back into the program to guide the interventions in a more targeted, efficient manner. To date, few published papers have used machine learning techniques to predict maternal or child outcomes in SSA (26, 30–37), only one of which focused on facility-based delivery (31). Of note, this latter example from Ethiopia used national survey data and not data captured on individuals enrolled in a maternal health program.

To address these gaps, we applied several machine learning techniques to predict health facility delivery among pregnant women enrolled in the maternal health program, *Uzazi Salama*, in Zanzibar, Tanzania (38). We used existing program data, captured at the time of enrollment by CHWs equipped with a smartphone application, to build these models. In this paper, we first provide an overview of the *Uzazi Salama* program and data collection followed by rationale for choice of variables and machine learning models. We conclude with a discussion on the benefits of integrating prediction models within *Uzazi Salama* or similar maternal health programs with the goal of improving rates of facility delivery.

## Materials and methods

### Program overview

The *Uzazi Salama* (“Safer Deliveries”) program operated between 2016 and 2019 in Unguja and Pemba, the two main islands of Zanzibar. In January 2020, the program was adopted by the Government of Zanzibar as the national community health program, expanded to provide additional postpartum and child health services, and was renamed *Jamii ni Afya* (“Communities are Health”). The goal of the *Uzazi Salama* program was to increase the number of women delivering in a health facility by providing birth planning, counseling, and care referrals via in-person home visits with a community health volunteer. Henceforth, we will refer to community health volunteers as CHWs to be consistent with the literature. CHWs provided three scheduled health promotion home visits prior to delivery. CHWs used a program-specific cell phone application to guide the encounter, which also served as a real-time data collection tool. Further details regarding the Uzazi Salama program are available in Supplementary Material.

### Study population

We included women enrolled in *Uzazi Salama* between 1 January 2017–30 June 2019 with a recorded delivery in this analysis. Women had to deliver at a health facility or at home to be included. Women who delivered on the way to the facility and women with no birth location documented were excluded. A total of 38,787 women met the inclusion criteria and were considered in the analysis.

### Key variables

The models presented here depend on individual- and community-level variables. All analyses are based *strictly* on information collected by CHWs prior to or during the enrollment visit. The considered variables are as follows: Woman's demographic characteristics included district of residence, age, and the following six socioeconomic indicators: whether all children are currently living, electricity in home, drinking water source, roof material, floor material and education level. CHWs collected these six socioeconomic indicators beginning in March 2017 during in-person home visits occurring one week postpartum. Although this information was collected later, it is assumed that the answers apply to the enrollment visit and in the new national program this data is being collected at the first visit. Self-reported clinical characteristics included details on (a) comorbidities (HIV status, cardiac disease, diabetes, high blood pressure, sickle cell anemia) and (b) past pregnancies as relevant (parity category (0, 1–2, 3–4, 5–7, 8+), previous delivery location, eclampsia, perineal tear, placenta previa, prolonged labor, retained placenta, vacuum, cesarean section, spontaneous abortions or stillbirths).

Current pregnancy characteristics included whether it was a twin pregnancy, whether the baby was larger than average (macrosomia), and the amount of money the family had currently saved for the delivery. It also included the total number of antenatal care (ANC) visits prior to the enrollment visit. If the first ANC visit took place prior to enrollment, information on the occurrence of convulsions, abdominal pain, bleeding, headache or difficulty breathing was recorded, along with whether the mother was charged a fee and whether the first ANC visit was a “negative experience”, defined as having both a privacy interruption and a wait time of more than 3 h. We also considered if the woman was referred back to a health facility by the CHW at the first visit and, if so, the reason for referral. Program-associated characteristics, also collected at the enrollment visit, included partner permission to deliver in a health facility (which required the partner to be present at one of the CHW visits, else not received), estimated gestational age at enrollment, type of recommended delivery facility, taxi price to delivery facility as a distance proxy, whether the woman had her reproductive and child health card available during the visit. Name of the recommended delivery facility was included as a proxy for perception of quality and to account for regional differences in the patient population.

Finally, community-level characteristics were created for inclusion in the model. For each woman, the following variables were calculated based on program-level information up to the time of the enrollment visit: cumulative level of experience of the woman's CHW measured in 6-month increments, rate of facility delivery in mother's shehia (local area), and rate of facility delivery among women with the same CHW*.* These were derived from the original data such that the rates were only based on women who delivered *prior* to the enrollment date for the woman of interest, including those enrolled in the program prior to 1 January 2017. Women living in the same shehia sometimes had the same CHW, resulting in some correlation between these variables.

### Data preprocessing

Some women were missing information on savings (10%) and the six socioeconomic indicators (33%) because these variables were added to the smartphone app after the start of the program and because information was collected at a specific postpartum home visit, which some women may have missed. Data availability was high (>97%) across all other variables. Missing values in nominal variables were imputed with the most frequent category. *K*-Nearest Neighbor (KNN) imputation (39), which uses observations from *K* “similar” women in the dataset to impute a plausible value for the woman with a missing variable, was used to impute all other missing values. *K* = 1 was used for ordinal variables and *K* = 5 for all other variables.

**Figure 1:**
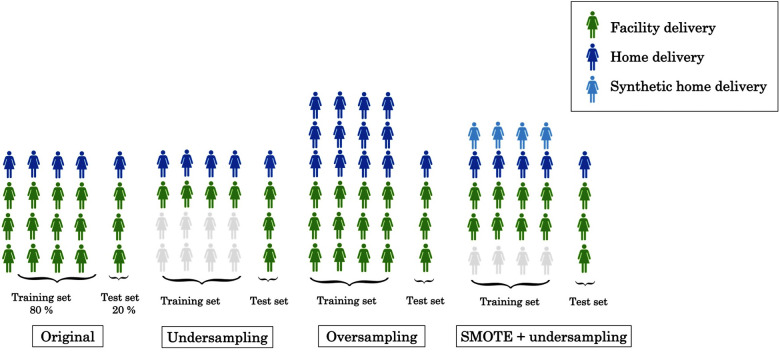
Illustration of training set undersampling, oversampling and SMOTE with undersampling.

We created two datasets: 80% of the women were randomly selected, stratified by home or facility delivery, for the *training set* to be used for model development and the remaining 20% were used as a *test set* after completion of model development. In both the training and test sets, 77% of women had delivered at a health facility, creating an outcome imbalance which could cause some prediction models to prioritize predicting all or most observations as facility deliveries. Three different techniques were employed to address this imbalance: undersampling, oversampling and a synthetic minority oversampling technique (SMOTE) (40). First, we undersampled from the facility deliveries in the training set by randomly selecting a subset of the same size as the number of home deliveries, as illustrated in Figure 1. This approach does not make full use of the information available but avoids the issue of poorer classification of home deliveries directly due to the imbalance of delivery facility types.

Second, we used oversampling from the minority class (home deliveries), whereby some observations occur multiple times in the training set. This increases the risk of overfitting but makes full use of the information in the sample. Finally, we also approached the imbalance issue using SMOTE, which creates synthetic records that are “similar” to actual records from women who delivered at home (see Section 4.2 of (40) for further technical details). To avoid relying too heavily on synthetic records, without compromising on the outcome balance, we simultaneously undersampled from the facility deliveries.

### Statistical modeling

Prior to model development, we summarize the characteristics of women captured in the training dataset and use the chi-square statistic to identify the ten factors most associated with facility delivery. Continuous features (age, gestational age, taxi price to delivery facility, delivery savings, payment fee at first ANC visit, rate of facility delivery in shehia, rate of facility delivery among women with same CHW) were discretized for this calculation, but were left continuous for the prediction modeling.

We then used four different models, comprising two parametric and two non-parametric approaches, with varying levels of regularization, given that prior knowledge about the exact relationship between the outcome and the predictors was very limited. As a baseline model, we used multivariate logistic regression, assuming a linear relationship between the covariates and the log-odds of facility delivery. We also considered a logistic model using LASSO regularization (41), a means of feature selection to shrink the coefficients of “less predictive” covariates towards 0.

In addition, two non-parametric models were used: a random forest, which calculates an average prediction across a large set of decision trees based on bootstrapped samples and random subsets of features, and an artificial neural network, which consists of layers of neurons that each look specifically at certain features of data. The tree depth for the random forest was selected using cross validation to reduce the risk of overfitting to the training set and making insufficient use of the information available in the data.

While the logistic and the regularized logistic models are easily interpretable and the latter provides insight into variable importance (41), the random forest model is more flexible and may better capture complex non-linear relationships between the variables and the outcome (29) while offering a measure of relative variable importance (42). Deep learning models typically offer additional flexibility, but require larger sample sizes, are more computationally expensive, and offer limited interpretability on variable importance (43).

### Model evaluation

We used multiple metrics to assess how well the test set predictions matched the true delivery locations. The baseline metric used was overall test set accuracy, the percent of women in the test set that were correctly classified. Given the skewed test set, with only ∼23% being home deliveries, the individual performance on the minority and majority class was also measured using true positive rate (sensitivity), which measures the proportion of home deliveries that are classified correctly and true negative rate (specificity), which measures the proportion of facility deliveries that are classified correctly. Finally, the area under the receiver operating characteristic curve (AUC) is a measure that jointly accounts for true and false positives, thus offering an appropriate method for comparison among models with similar performance across the other metrics. We computed the chi-square statistic to estimate which predictors best discriminated between facility and home deliveries. All analysis was conducted using R version 3.6.1 and Python packages scikit-learn 0.23.1 and tensorflow 2.4.1. The code used for model training and testing is available in the GitHub repository almafredriksson/facility-delivery.

## Results

Among the 31,435 women in the training set, 76.6% delivered at a health facility and 23.4% delivered at home after taking part in Uzazi Salama. A summary of the demographic, programmatic and self-reported clinical characteristics for the full dataset is presented in Tables 1, 2. We find that the 10 predictors that *individually* best discriminate between home and facility deliveries are the following, in order of magnitude of association:

(1) Previous delivery location
(2) Rate of facility delivery among women with the same CHW
(3) Rate of facility delivery in shehia
(4) Name of recommended delivery facility
(5) Residential district
(6) Parity
(7) Education level
(8) Floor material
(9) Access to electricity in home
(10) Payment fee charged at first ANC visit

**Table 1:** Demographic characteristics (*n* = 38,787).

Variable	*N* (%)
**Overall**	38787 (100%)
**Delivery location**	
Health facility	29679 (76.5%)
Home	9108 (23.5%)
**District**	
Kaskazini A	5799 (15.0%)
Kaskazini B	2831 (7.3%)
Kati	3529 (9.1%)
Magharibi	4853 (12.5%)
Kusini	1861 (4.8%)
Mkoani	6135 (15.8%)
Wete	3917 (10.1%)
Micheweni	4471 (11.5%)
Chake Chake	5391 (13.9%)
**Age of mother**	
10–20	5730 (14.8%)
21–30	22406 (57.9%)
31–40	9710 (25.1%)
41+	868 (2.2%)
**Parity**	
0	8916 (23.0%)
1	6648 (17.2%)
2–4	14980 (38.7%)
5+	8168 (21.1%)
**Previous delivery location**	
At home/in community	7946 (20.5%)
On the way to health facility	338 (0.9%)
Health facility	21514 (55.6%)
No previous delivery	8916 (23.0%)

**Table 2:** Programmatic and self-reported clinical characteristics (*N* = 38,787).

Variable	*N* (%)
**Overall**	38787 (100%)
**Gestational age at enrollment (weeks)**	
0–10	1599 (4.1%)
11–20	15231 (39.3%)
21–30	18612 (48.0%)
31–40	3331 (8.6%)
**Partner permission to enroll**	
Yes	16881 (43.5%)
No	21906 (56.5%)
**Taxi price to delivery facility (TZS)**	
0–10,000	14101 (36.6%)
10,001–20,000	13573 (35.3%)
20,001–30,000	7280 (18.9%)
30,000+	3544 (9.2%)
**CHW experience level (months)**	
0–6	8567 (22.3%)
7–12	9176 (23.9%)
13–18	8779 (22.8%)
19–24	7394 (19.2%)
25+	4522 (11.8%)
**HIV status**	
Positive	443 (1.2%)
Negative	37367 (96.3%)
Unknown	977 (2.5%)
**Cardiac disease**	
Yes	52 (0.1%)
No	38662 (99.9%)
**High blood pressure**	
Yes	132 (0.3%)
No	38582 (99.7%)

**Figure 2:**
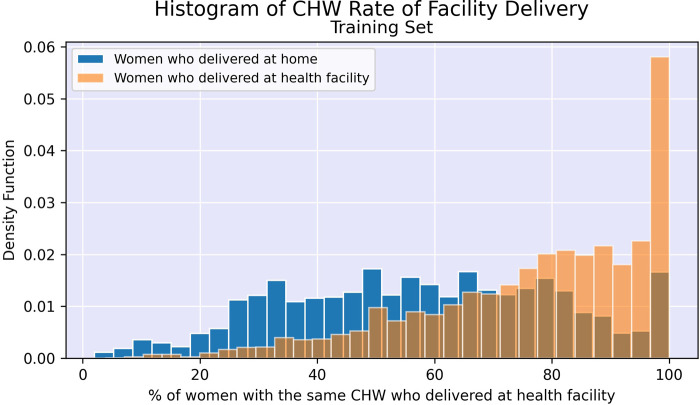
Histogram of facility delivery rates among women with the same community health worker.

**Figure 3:**
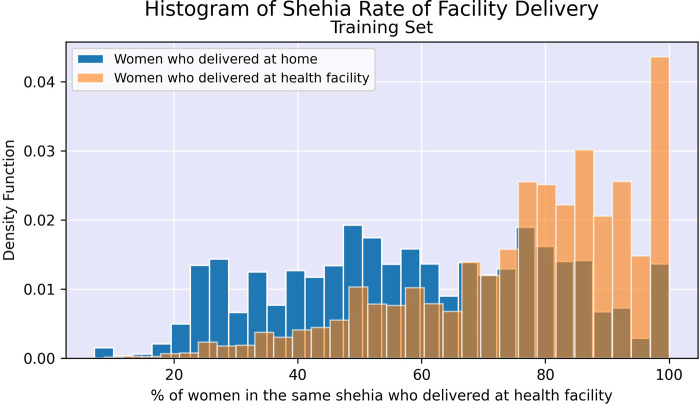
Histogram of facility delivery rates among women in the same shehia (local area).

Figures 2, 3 illustrate the difference in distribution between women who delivered in a facility and women who delivered at home for key continuous predictors above. Further details regarding test statistics for all top variables and figures visualizing the difference in socioeconomic distribution between women who delivered at a health facility and women who delivered at home are available in Supplementary Material.

### Undersampling

After performing undersampling, the training set consisted of 14,572 women; 7,286 women from each outcome class. The logistic regression model, its regularized counterpart and the random forest model achieve an overall classification accuracy of 72%–74% on the test set (see Table 3). While these three models have a slight difference in accuracy across the two outcome classes, the neural network shows a major performance gap, where facility deliveries are more accurately predicted than home deliveries. The random forest model shows the most promising performance on the home deliveries with correct classification in more than 74% of the cases. The associated variable importance measure, reflecting how often a variable is used in the condition that splits a tree branch into two, suggested that the rate of facility delivery among women in the same shehia or with the same CHW are key predictors. As previously noted, there is some correlation between these variables as women in the same shehia may have the same CHW.

**Table 3:** Model performance on test set by training set type.

Training set	Classifier	True positive rate^a^	True negative rate^b^	Overall accuracy	AUC
Undersampled *N* = 14,572	Logistic	71.7%	74.0%	73.5%	0.801
	Regularized Logistic	71.0%	74.5%	73.7%	0.799
	Random Forest	74.4%	71.8%	72.4%	0.800
	Neural Network	56.1%	80.3%	74.6%	0.744
Oversampled *N* = 47,486	Logistic	71.1%	74.5%	73.7%	0.802
	Regularized Logistic	71.0%	74.6%	73.8%	0.802
	Random Forest	71.1%	73.8%	73.1%	0.799
	Neural Network	75.1%	65.9%	68.1%	0.772
SMOTE with minor undersampling *N* = 30,000	Logistic	71.8%	74.0%	73.5%	0.801
	Regularized Logistic	71.1%	74.5%	73.7%	0.802
	Random Forest	68.6%	76.5%	74.6%	0.798
	Neural Network	58.9%	80.8%	75.7%	0.778

^a^ Proportion of home deliveries that are classified correctly.

^b^ Proportion of facility deliveries that are classified.

### Oversampling

After performing oversampling, the training set consisted of 47,486 women; 23,743 women who had delivered at a facility, 7,368 unique observations from women who delivered at home, and 16,735 observations from women who delivered at home that were sampled from the 7,368 unique observations. The neural network shows a promising true positive rate and correctly identifies more than 75% of home deliveries (see Table 3). The logistic and regularized logistic models both achieve an overall accuracy of close to 74%. The similar performance across the two logistic models suggests that little or no feature selection occurred.

### SMOTE with minor undersampling

After performing SMOTE with minor undersampling, the training set consisted of 30,000 women; 15,000 women who had delivered at a facility, 7,368 unique observations from women who delivered at home, and 7,632 synthetic observations similar to women who delivered at home. The unregularized logistic and regularized logistic regression models trained on synthetic records performed well with true positive rates just below 72% (see Table 3), but generally predicted facility deliveries more accurately than home deliveries.

Further details about the false positive rate and false negative rate of each model are available in Supplementary Material.

## Discussion

Our paper is one of few using machine learning approaches to predict facility delivery in SSA. Across varying modeling and balancing techniques, we found the accuracy to be 68%–77%, with slightly higher accuracy when predicting facility delivery versus home delivery. One other paper has previously shown the utility of these types of models and successfully predicted skilled birth attendance in Ethiopia using retrospectively collected survey data (31). The model we present operates on program data collected at enrollment, including community-level factors, which enables prospective implementation and integration into existing maternal health programs. It could also be used in real-time to assess the risk of home delivery and provide tailored intervention. We found education, age and parity to be important, which corroborates findings from other papers (9–14). However, we also found community-level variables to be highly predictive, highlighting their importance for inclusion in the prediction models.

For all but two combinations of modeling and balancing techniques, the performance was higher when predicting facility delivery, which is consistent with the fact that all the models based on oversampling and synthetic records have less real information about home deliveries available regardless of the balanced training set. Importantly, the classification used in this paper is optimized for overall prediction accuracy, but in practice, the classification thresholds could be altered manually to increase or reduce the sensitivity and specificity of the algorithm according to maternal health program priorities and the cost of specific interventions used. Across the undersampled and the oversampled training set, the random forest model and the neural network respectively obtain the highest true positive rate or, equivalently, shows the strongest performance on home deliveries (74.4% and 75.1% respectively). The former achieves this without compromising noticeably on the performance for facility deliveries. For the partly synthetic training set, the logistic regression model achieves the highest classification accuracy on the home deliveries at 71.8%. Among these three candidate models, the undersampled random forest is the only one to show strong performance on the home deliveries, which we are particularly interested in identifying, without noticeably sacrificing performance on the facility deliveries. This strong performance of the non-parametric random forest model relative to the parametric methods suggests that there may be non-linear relationships present that the traditional parametric models may not fully capture.

While the training data is specifically obtained from *Uzazi Salama*, the framework is applicable in other contexts and the selection of input features can easily be adapted to match data availability and other outcomes, both within and beyond maternal health. The insights obtained here will be leveraged to predict delivery location for pregnant women who are enrolled in the *Jamii ni Afya* program. We hypothesize that the prediction accuracy will be improved as socioeconomic data will be collected for all individuals. Extensions of this work include updating predictions after additional data is collected at future CHW home visits, which would allow for multi-stage program tailoring.

There are several limitations to our findings, including missing data, which is a common issue in these contexts. In the *Uzazi Salama* data, we had missingness in several variables, primarily among the socioeconomic indicators. We used the KNN algorithm to impute values, given that it generally performs well compared to similar techniques and the risk of model misspecification is low, as it is fully non-parametric (40). However, applying it to a dataset with a vast number of predictors increases the risk of relying on features with limited relevance to the outcome, which can add randomness to the distance measure used to select neighbors and subsequently reduce the quality of the imputation (44). We further recognize that although several socioeconomic indicators were included in the analysis, there may be nuances of each woman's socioeconomic status that have not been fully captured.

Similar to most maternal health programs, *Uzazi Salama* also experienced loss to follow-up. Approximately 14% of enrollees did not have a delivery outcome recorded and were excluded from the analysis, as we did not feel comfortable imputing these. We hypothesize that women who are lost to follow-up have either traveled to another location, such as a family home, to deliver and CHWs were not able to reach them or they are hesitant to tell their CHW that they delivered at home. However, as this study does not claim causality, the main concern of this potential data bias is primarily reduced prediction accuracy rather than confounding.

## Conclusion

We compared four machine learning models and three sampling techniques to display the utility of using routinely collected maternal health program data to predict delivery location. Our models correctly captured the delivery location for 68%–77% of the women in our test set, with slightly higher accuracy when predicting facility delivery versus home delivery. We demonstrate that prediction models can effectively be used to obtain a “real-time” prediction of the delivery location for new maternal health program enrollees. Embedded in a cell phone application, this model can be leveraged to immediately alert the CHW to individuals at particularly high risk of delivering at home, allowing for provision of extra support or further visits, which has potential to improve health outcomes for both mothers and children. While our training data originates from the *Uzazi Salama*, the framework is applicable in other contexts and the selection of input features can easily be adapted to match data availability and other outcomes, both within and beyond maternal health.

## Data Availability

The data analyzed in this study is subject to the following licenses/restrictions: The dataset presented in this article is not readily available as it contains personal health data and is subject to data privacy regulation. Requests to access these datasets should be directed to Tracey Li, tli@d-tree.org.
