# Supplementary material for: Machine learning for maternal health: Predicting delivery location in a community health worker program in Zanzibar
Source: Front Digit Health. 2022 Aug 17;4:855236. doi: 10.3389/fdgth.2022.855236 (PMC9428344; doi:10.3389/fdgth.2022.855236)
Supplement: Supplementary file 1 [file Data_Sheet_1_v2.pdf]

Supplementary material for

Machine learning for maternal health: predicting delivery location in a community health worker program in Zanzibar

Alma Fredriksson[1], Isabel Fulcher[1,2], Allyson Russell[3], Tracey Li[3], Samira Seif[3], Bethany Hedt-Gauthier[1,2]

[1]Department of Biostatistics, Harvard T.H. Chan School of Public Health, Boston, USA.
[2]Department of Global Health and Social Medicine, Harvard Medical School, Boston, USA.
[3]D-tree International, Dar es Salaam, Tanzania.

Acknowledgements
We would like to thank the Zanzibar Ministry of Health for their collaboration in the *Uzazi Salama* and *Jamii ni Afya* programs.

Conflict of interest
The authors declare that the research was conducted in the absence of any commercial or financial relationships that could be construed as a potential conflict of interest.

CONTENT

1. **Further details regarding the Uzazi Salama program**
2. **Further details regarding the socioeconomic predictors**
3. **Further details regarding chi-square test to identify key predictors**
4. **Further model performance metrics**

1. Further details regarding the Uzazi Salama program

Further detail regarding the distribution of new monthly enrollments, deliveries and program exits in the Uzazi Salama in Zanzibar is illustrated in *Supplementary Fig. 1*.

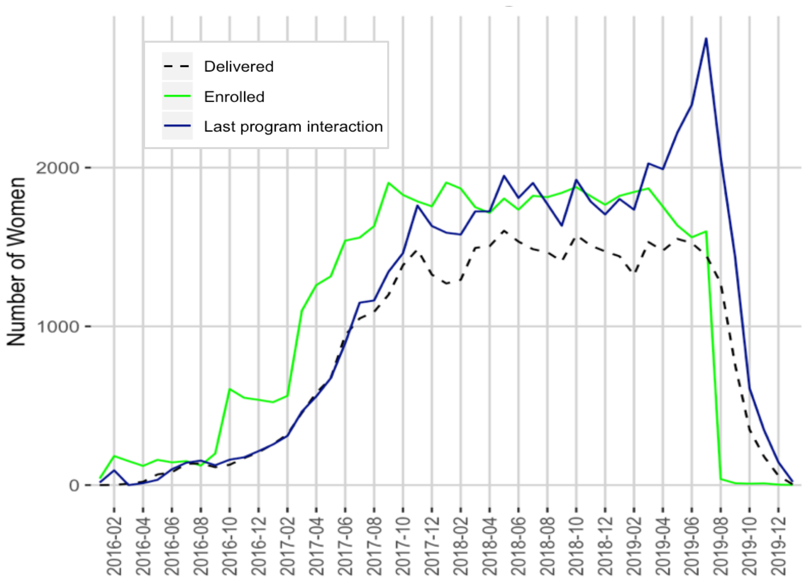

Supplementary Figure 1. *Distribution of new monthly enrollments, deliveries and program exits in the Uzazi Salama in Zanzibar.*

2. Further details regarding socioeconomic predictors

The analysis of each predictor's ability to individually discriminate between women who deliver in a health facility and women who deliver at home, presented in the *Results* section, suggests that socioeconomic indicators are important factors. *Supplementary Fig. 2* below illustrates the difference in socioeconomic distribution between women who deliver in a health facility and women who deliver at home.

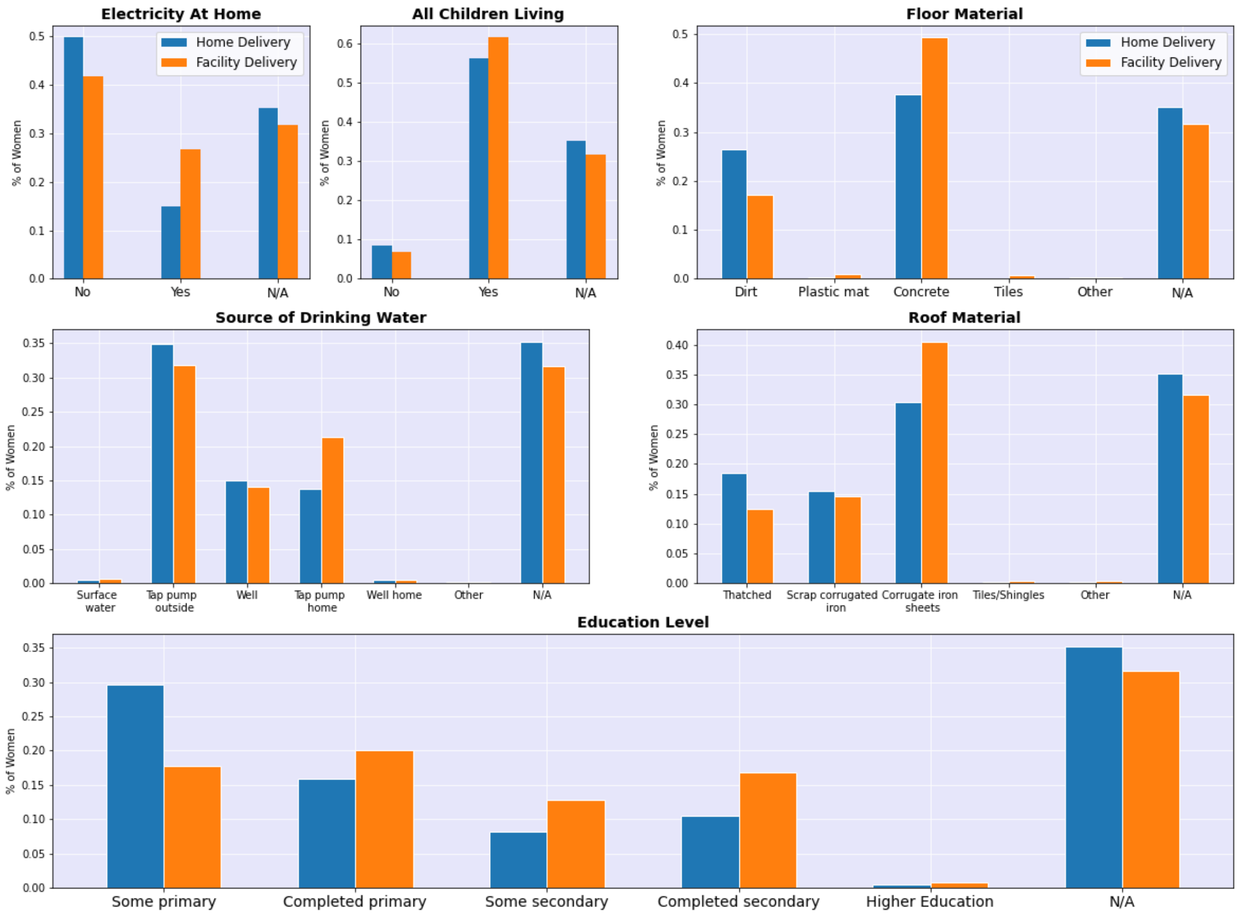

Supplementary Figure 2. Distribution of facility and non-facility deliveries across socioeconomic indicators

3. Further details regarding chi-square test to identify key predictors

Further detail regarding the outcome distribution across key predictors and their corresponding χ^2-statistic is provided in *Supp. Table 1.*

Supplementary Table 1. Demographic and programmatic characteristics by delivery location (N=38,787)

VARIABLE	HEALTH FACILITY DELIVERY<br>N (PROPORTION OF TOTAL WOMEN IN CATEGORY)	χ^2 STATISTIC	P-VALUE
**Overall**	29679 (76.5%)		
**Previous delivery location**		4580.1	<0.001
At home/in community	3841 (48.3%)		
On the way to health facility	202 (59.8%)		
Health facility	17853 (83.0%)		
No previous delivery	7730 (86.7%)		
**Rate of facility delivery among women with same CHW**		4407.7	<0.001
Q1 (2 - 58.1%]	4796 (54.1%)		
Q2 (58.1 – 77.6%]	6729 (75.9%)		
Q3 (77.6 - 91.3%]	7726 (86.8%)		
Q4 (91.3 - 100%]	8201 (92.7%)		
**Rate of facility delivery among women in same shehia**		4265.1	<0.001
Q1 (7.2 - 58.3%]	4918 (54.1%)		
Q2 (58.3 – 77.8%]	6922 (76.3%)		
Q3 (77.8 - 88.7%]	7742 (85.7%)		
Q4 (88.7 - 100%]	8350 (92.2%)		
**Name of Delivery Facility**<br>List of specific facilities omitted due to length.		2370.6	<0.001
**District**		2298.4	<0.001
Kaskazini A	4333 (74.7%)		
Kaskazini B	2458 (86.8%)		
Kati	2874 (81.4%)		
Magharibi	4291 (88.4%)		
Kusini	1791 (96.2%)		
Mkoani	3686 (60.1%)		
Wete	3015 (77.0%)		
Micheweni	2910 (65.1%)		
Chake Chake	4321 (80.2%)		
**Parity**		954.9	<0.001
0	7730 (86.7%)		
1	5183 (78.0%)		
2-4	11241 (75.0%)		
5+	5470 (67.0%)		
**Education level**		867.9	<0.001
Some primary	5291 (66.2%)		
Completed primary	5939 (80.6%)		
Some secondary	3806 (83.5%)		
Completed secondary	4982 (83.9%)		
Higher education	245 (85.1%)		
**Floor material**		623.3	<0.001

Dirt	5093 (67.6%)		
Plastic mat	259 (91.8%)		
Concrete	14640 (81.2%)		
Tiles	198 (91.2%)		
Other	73 (79.3%)		
**Electricity in home**		508.6	<0.001
Yes	7908 (85.4%)		
No	12355 (73.2%)		
**Fee at first ANC visit (shilling)**		414.8	<0.001
Q1 (0 – 1,000]	2917 (67.6%)		
Q2 (1,000 – 2,000]	1206 (82.3%)		
Q3 (2,000 – 3,000]	3241 (80.9%)		
Q4 (3,000 – 10,000]	4833 (83.5%)		

4. Further model performance metrics

Table 3 presents the true positive rate, true negative rate, overall accuracy and AUC of each of the trained models. *Supplementary Table 3* provides additional detail on the false positive rate, women predicted to deliver at home who actually delivered in a health facility, and false negative rate, women who were predicted to deliver at a health facility, but delivered at home.

Supplementary Table 3. Additional metrics for model performance on test set by training set type.

TRAINING SET	CLASSIFIER	FALSE POSITIVE RATE	FALSE NEGATIVE RATE
**Undersampled N=14,572**	Logistic	28.3%	26.0%
	Regularized Logistic	29.0%	25.5%
	Random Forest	25.6%	28.2%
	Neural Network	44.9%	19.7%
**Oversampled N = 47,486**	Logistic	28.9%	25.5%
	Regularized Logistic	29.0%	25.4%
	Random Forest	28.9%	26.2%
	Neural Network	24.9%	34.1%
**SMOTE with minor undersampling N = 30,000**	Logistic	28.2%	26.0%
	Regularized Logistic	28.9%	25.5%
	Random Forest	31.4%	23.5%
	Neural Network	41.1%	19.2%
